# Supplementary material for: Human neutralizing antibodies target a conserved lateral patch on H7N9 hemagglutinin head
Source: Nat Commun. 2024 May 27;15:4505. doi: 10.1038/s41467-024-48758-4 (PMC11130183; doi:10.1038/s41467-024-48758-4)
Supplement: Supplementary file 1 — Supplementary Information [file 41467_2024_48758_MOESM1_ESM.pdf]

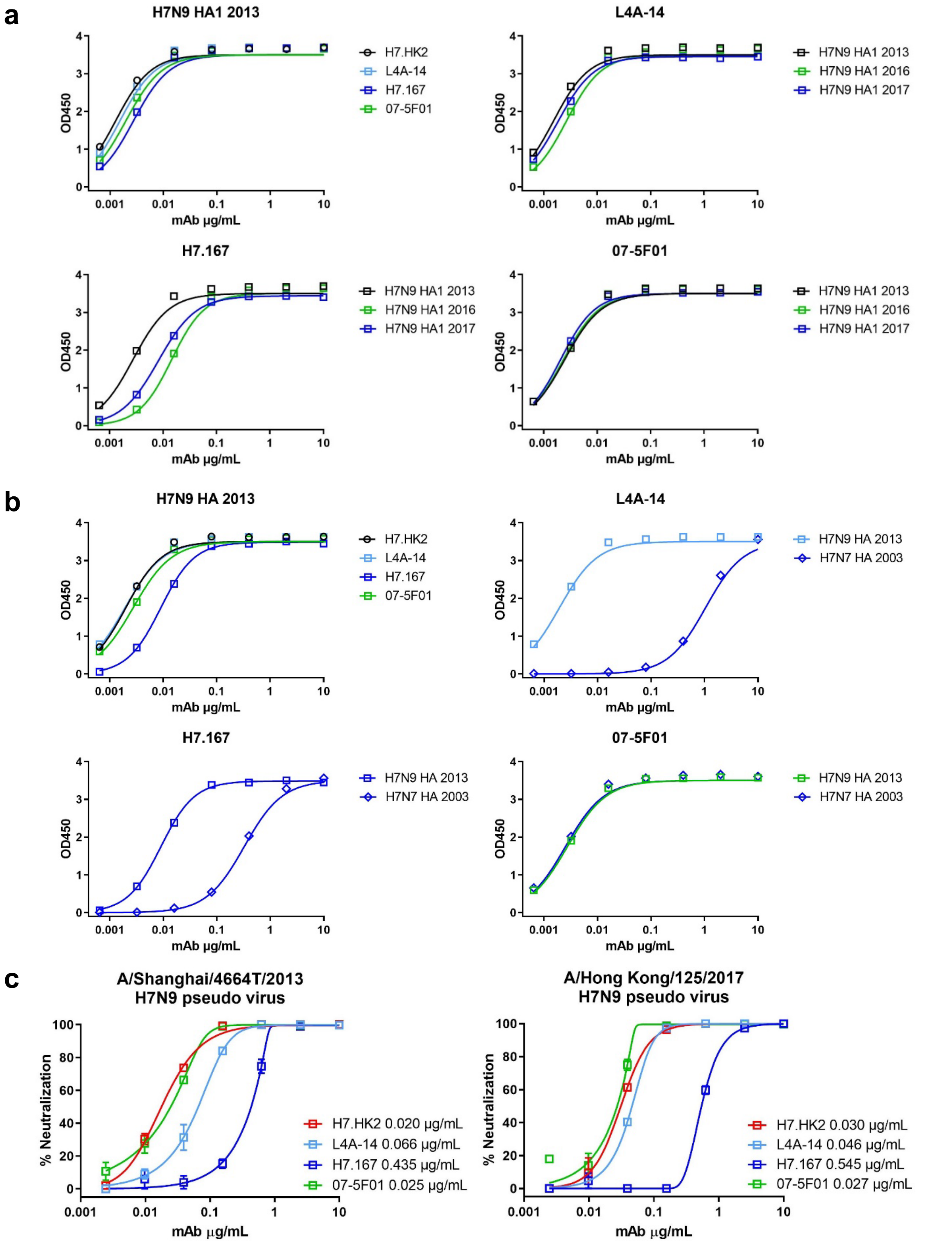

Supplementary Fig. 1 Comparisons of human H7N9 mAbs *in vitro*. a ELISA binding curves of the indicated mAbs to H7N9 HA1 from A/Shanghai/2/2013, A/Guangdong/17SF003/2016, and A/Hong Kong/125/2017. **b** ELISA binding curves of the indicated mAbs to H7N9 HA based on A/Shanghai/2/2013 and H7N7 HA based on A/Netherlands/219/2003. **c** Neutralization curves of the indicated mAbs against the 2013 and 2017 H7N9 pseudo viruses in MDCK cells. Data shown are mean ± SEM, with source data provided in a Source Data file. Similar results have been independently reproduced at least once.

Heavy Chain V-gene

```
            -------------FR1-------------- CDR H1-----FR2------______CDR H2______--------------FR3---------------
                     10        20        30          40        50         60        70        80 ABC       90
                      |         |         |           |         |          |         |         |            |
IGHV4-59    QVQLQESGPGLVKPSETLSLTCTVSGGSIS  SYYWSWIRQPPGKGLEWIGYIYYSGSTN YNPSLKSRVTISVDTSKNQFSLKLSSVTAADTAVYYC
H7.HK1      QVQLQESGPGLVKPSETLSLTCSVSGGSIN  SYYWTWIRQPPGKGLEWVGYIYHSGSTS YNPSLKSRITISVAPSKNHFSLELTSMTAADTAVYYCAR
H7.HK2      QVQLQGSGPGLLRPSETLSLTCSVSGVSIN  SYYWSWVRQPPGKALEWIGYIYYSGNTN YNPSLESRVTISVDRSKNQFSLKMTSVTAADTARYFCAR

IGHV7-4-1   QVQLVQSGSELKKPGASVKVSCKASGYTFT  SYAMNWVRQAPGQGLEWMGWINTNTGNPTYAQGFTGRFVFSLDTSVSTAYLQICSLKAEDTAVYYC
H7.HK3      QVQLVQSGSELKRPGASVKVSCRASGYTFT  SYTINWVRQAPGQGLEWMGWINTSTGDPTYAQGFTGRFVFSLDTSVSTAYLEISRLKAEDTAVYYCAR

IGHV4-61    QVQLQESGPGLVKPSETLSLTCTVSGGSVSSGSYYWSWIRQPPGKGLEWIGYIYYSGSTN YNPSLKSRVTISVDTSKNQFSLKLSSVTAADTAVYYC
H7.HK4      QVQLQESGPGLVKPSETLSLTCTVSGGSVRSASYAWSWIRQPPGKGLEWIGDIYYSGTTN YNPSLKSRVTLSVDTAKNRFSLRLRSVTAADTAVYHCAR
```

Light Chain V-gene

```
            -----------FR1---------______CDR L1______------FR2------ CDR L2--------------FR3---------------
                     10        20      ABCDE  30         40        50        60        70        80      88
                      |         |              |          |         |         |         |         |       |
IGKV2-28    DIVMTQSPLSLPVTPGEPASISCRSSQSLLHSNGYNYLDWYLQKPGQSPQLLIYLGSNRASGVPDRFSGSGSGTDFTLKISRVEAEDVGVYYC
H7.HK1      DIVMTQSPVSLPVTPGEPASISCNSSQSLLHSNGYAHLDWYLQKPGQSPKLMIYLGLNRAFGVPDRFSGSGSGTDFTLKISRVEAEDVGVYYC
H7.HK2      DIVMTQSPLSLPVTPGEPASISCRSNQSLQHSNGYVHLDWYRQKPGQSPHLLIYLGFNRASGVPDRFSGGGSGTDFTLKISRVEAEDVGVYYC

IGKV1-5     DIQMTQSPSTLSASVGDRVTITCRASQSI     SSWLA WYQQKPGKAPKLLIYDASSLESGVPSRFSGSGSGTEFTLTISSLQPDDFATYYC
H7.HK3      DIQMTQSPSTLSASVGDRVTITCRASQSI     SSWLA WYQQKPGKAPKLLIYKASSLESGVPSRFSGSGSGTEFTLTISSLQPDDFATYYC

IGKV1-16    DIQMTQSPSSLSASVGDRVTITCRASQGI     SNYLA WFQQKPGKAPKSLIYAASSLQSGVPSRFSGSGSGTDFTLTISSLQPEDFATYYC
H7.HK4      DIQMTQSPSSLSASVGDRVTITCRASQGI     RNYLA WFQQKPGQAPKSLIFAASSLHTGVPSRFSGSGSGTDFTLTISSLQPEDFATYYC
```

CDR3

```
            ____CDR H3____----FR4----     ___CDR L3_----FR4---
            95 100ABC    103     110      90       98      106
            |    |         |       |       |        |        |
H7.HK1      LGGHGDYGSDY  WGQGTLVTVSS      MQALQTPFTFGPGTRVDIK
H7.HK2      QGIFGDYGSDY  WGPGTLVTVSS      MQGLQTPFTFGPGTTVDLK
H7.HK3      AFGLTVVRGGIVGVWGQGTTVTVSS     QQYNSYSQTFGQGTKVEIK
H7.HK4      ERYYYGSSGDFDY WGQGTLVTVSS     QHYNSYPPTFGQGTKLEIK
```

Supplementary Fig. 2 H7.HK mAb sequences. Protein sequences of the heavy and light chain variable regions of H7.HK mAbs are aligned to the putative germline V-genes at top, with amino acid substitutions in red, and in magenta for substitutions shared between the clonally related mAbs H7.HK1 and H7.HK2. Spaces are added to maintain alignment; framework regions (FR) and complementarity-determining regions (CDRs) are indicated based on the Kabat numbering and nomenclature. Highlighted in yellow are the mAb residues (paratopes of H7.HK1 and H7.HK2) contacting the H7 antigen. The putative N-linked glycosylation sites on the light chain CDR L1 of H7.HK1 and H7.HK2 and the heavy chain CDR H2 of H7.HK3 are underlined.

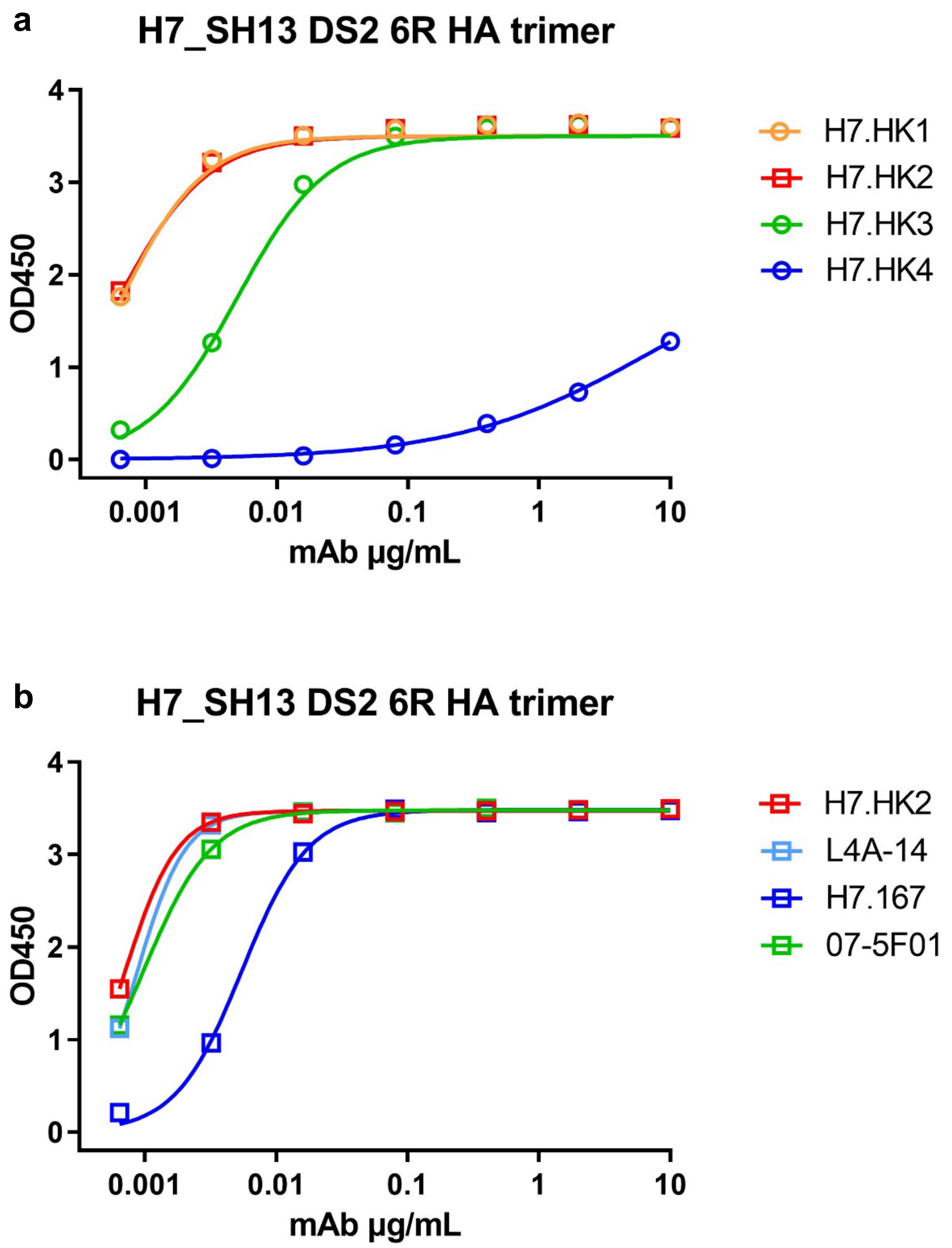

Supplementary Fig. 3 Human H7N9 mAbs binding to a soluble, disulfide-stabilized, fully cleaved H7 HA trimer H7 SH13 DS2 6R. a ELISA binding curves of the H7.HK mAbs to H7 SH13 DS2 6R HA trimer. **b** ELISA binding curves of previously reported mAbs to H7 SH13 DS2 6R HA trimer; the H7.HK2 mAb was included for comparison. Source data are provided in the Source Data file. Similar results have been independently reproduced at least once.

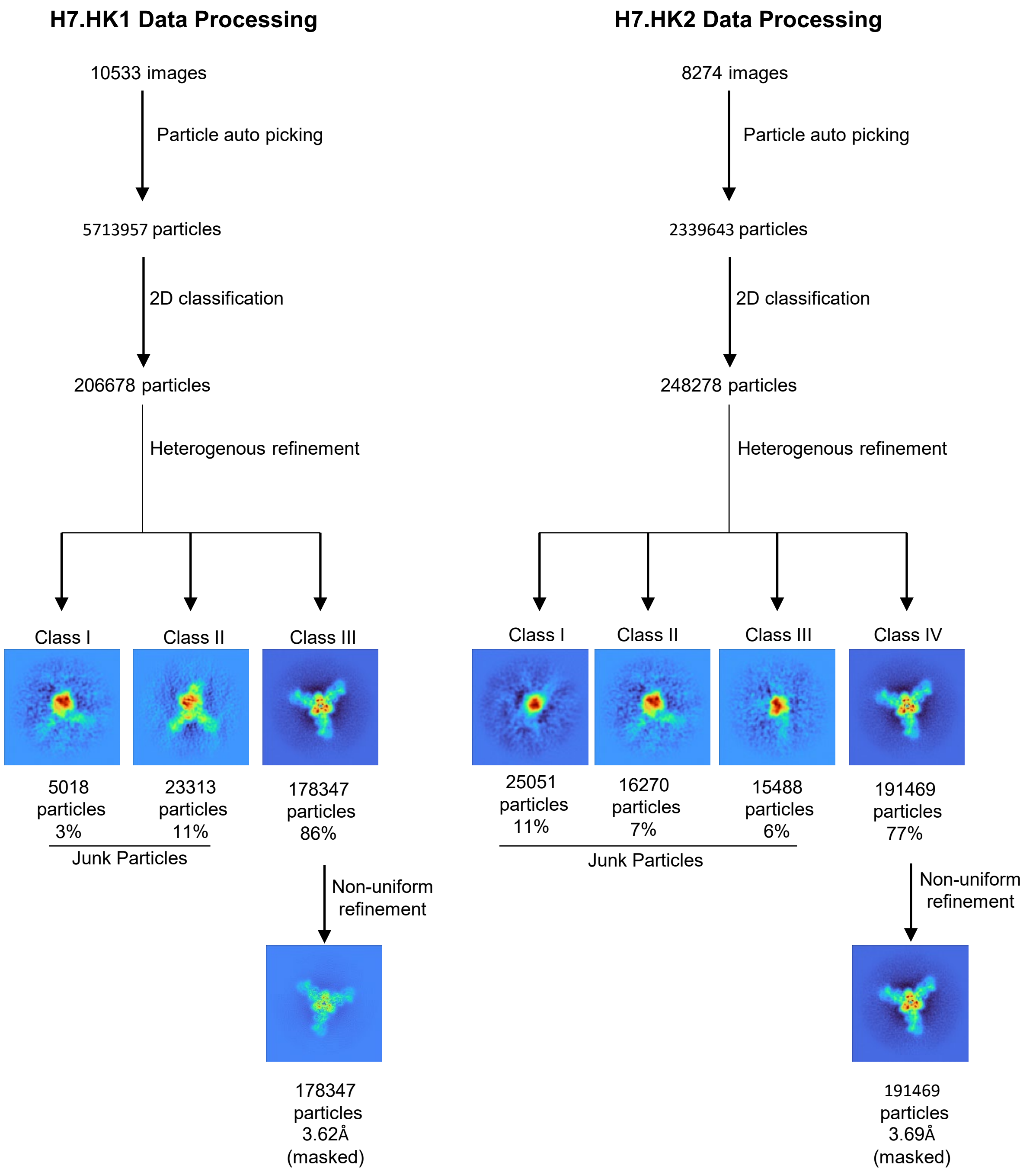

Supplementary Fig. 4 Cryo-EM data processing for H7.HK1 and H7.HK2 in complex with H7 SH13 DS2 6R HA trimer.

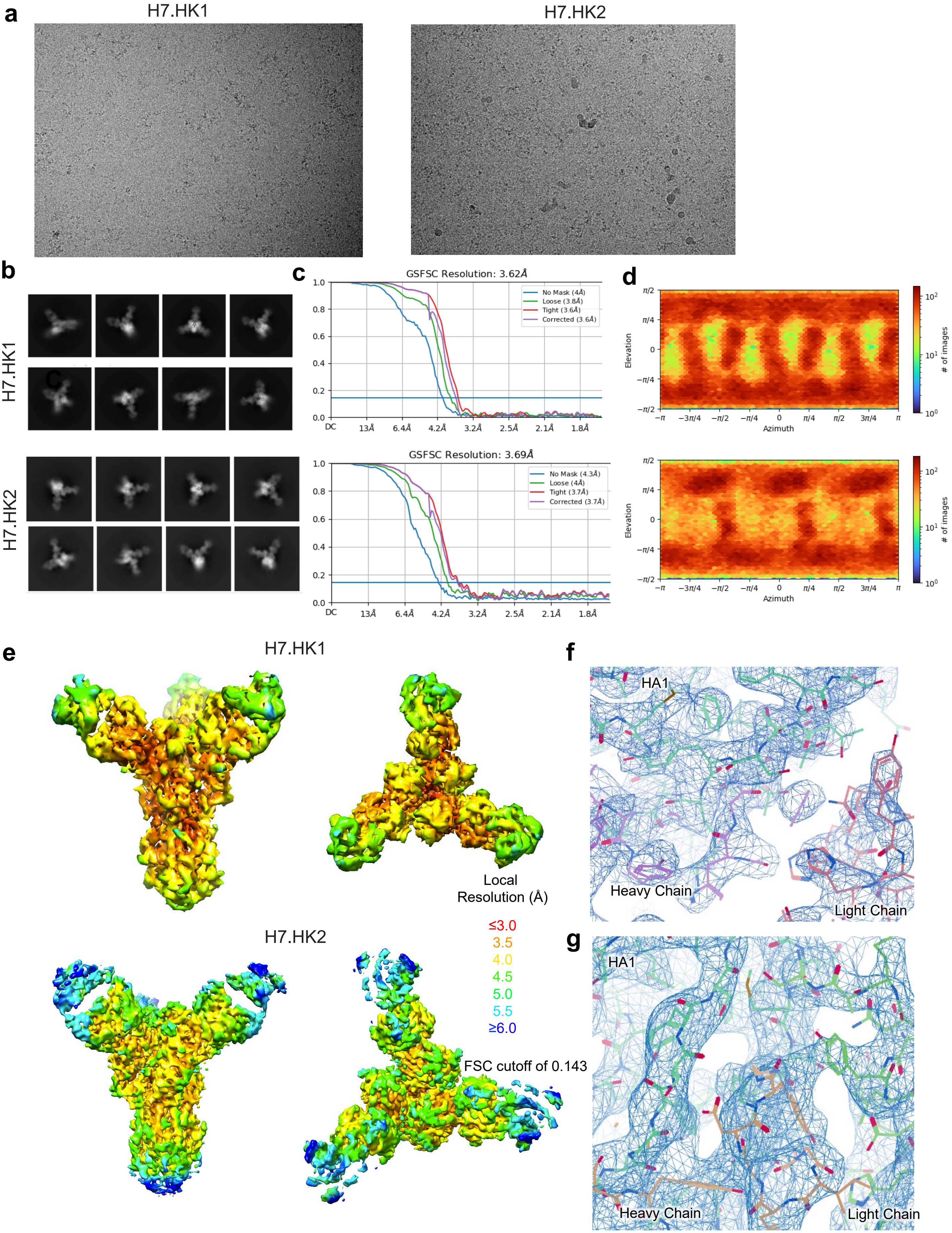

a
H7.HK1
H7.HK2
b
H7.HK1
H7.HK2
c
GSFSC Resolution: 3.62Å
No Mask (4Å)
Loose (3.8Å)
Tight (3.6Å)
Corrected (3.6Å)
GSFSC Resolution: 3.69Å
No Mask (4.3Å)
Loose (4Å)
Tight (3.7Å)
Corrected (3.7Å)
DC
13Å
6.4Å
4.2Å
3.2Å
2.5Å
2.1Å
1.8Å
d
Elevation
Azimuth
# of images
e
H7.HK1
H7.HK2
Local Resolution (Å)
≤3.0
3.5
4.0
4.5
5.0
5.5
≥6.0
FSC cutoff of 0.143
f
HA1
Heavy Chain
Light Chain
g
HA1
Heavy Chain
Light Chain

Supplementary Fig. 5 Cryo-EM details of H7.HK1 and H7.HK2 in complex with H7 SH13 DS2 6R HA trimer. a Representative micrograph of H7.HK1 and H7.HK2. **b** Representative 2D class averages of H7.HK1 and H7.HK2. **c** The gold-standard Fourier Shell Correlation (FSC) resulted in a resolution of 3.62 Å for the overall map of H7.HK1 and 3.69 Å for the overall map of H7.HK2. Non-uniform refinement with C3 symmetry was used for both reconstructions. **d** The orientations of all particles used in the final refinement are shown as a heatmap. **e** The local resolution of the final overall map is shown contoured at 0.0989 for both structures. Resolution estimation was generated through cryoSPARC using an FSC cutoff of 0.143. **f** Representative density is shown for the interface of H7.HK1 heavy chain, light chain, and H7 HA. **g** Representative density is shown for the interface of H7.HK2 heavy chain, light chain, and H7 HA.

a

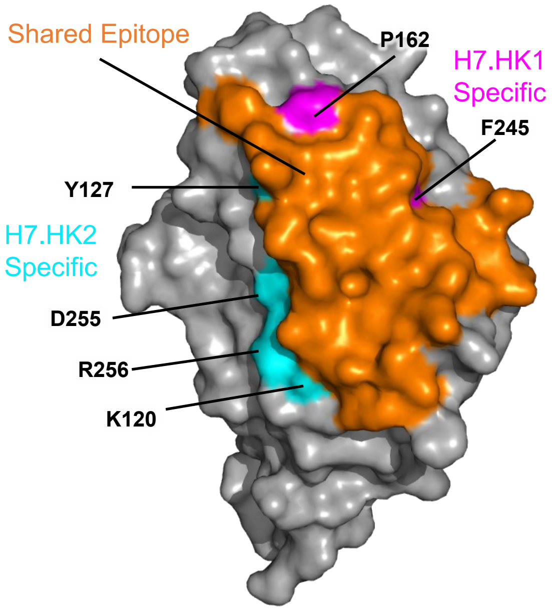

b

	H7.HK1 : **H7**	H7.HK2 : **H7**
Heavy Chain		Y52 : **Y168**
H Bonds	Y52 : **E121**	Y53 : **E121**
	R94 : **G124**	R94 : **G124**
	G99 : **S167**	G99 : **S167**
	D100 : **T126**	D100 : **T126**
	Y100a: **T165**	Y100a: **T165**
	Y100a: **S167**	Y100a: **S167**
	S100c: **T126**	S100c: **T126**
Heavy Chain Salt Bridge	H53 : **E121**	
Light Chain H Bonds	Y49 : **Q163**	Y49 : **T165**
		S56 : **G129**

c

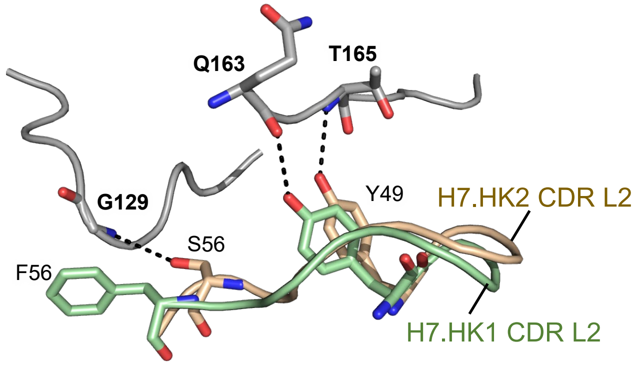

Supplementary Fig. 6 Comparison of H7.HK1 and H7.HK2 binding epitopes to H7. a Differences in the epitopes of H7.HK1 and H7.HK2. Majority of surface contacts are conserved, shown in orange. H7.HK1 specific surfaces are shown in magenta, and H7.HK2 specific surfaces are shown in cyan. **b** Hydrogen bonds and salt bridges formed by H7.HK1 and H7.HK2 with H7. **c** Differences in CDR L2 binding to H7 by H7.HK1 and H7.HK2 as a result of F56S substitution in H7.HK2. S56 forms an additional hydrogen bond with G129 of H7. Additionally, position of Y49 is shifted so that it forms a hydrogen bond with T165 for H7.HK2 instead of Q163 for H7.HK1.

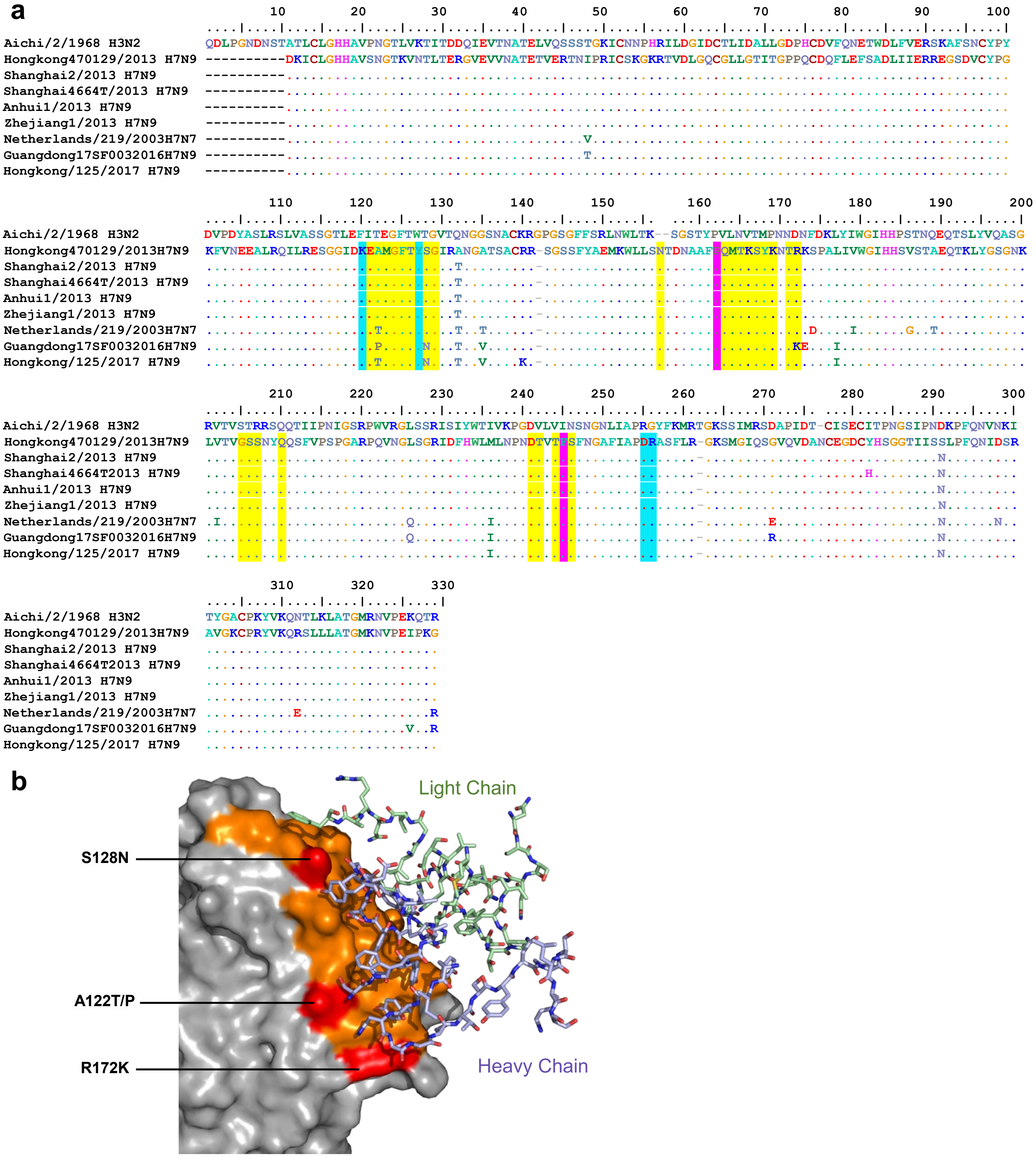

Supplementary Fig. 7 Antigenic drift of H7 HA1 in 2016-2017. a The A/Aichi/2/1968 H3N2 HA1 protein sequence is shown at top to indicate the H3 numbering of HA1. The H7 HA1 sequences from the indicated viral isolates are aligned to the 2013 Hong Kong H7N9 autologous isolate, with identical amino acids shown in dots. "-" depicts gap. Highlighted in yellow are the H7 contact residues (epitope) with both mAbs H7.HK1 and H7.HK2. H7.HK1 specific epitopes are in magenta; H7.HK2 specific epitopes are in cyan. **b** Surface presentation of the H7 HA1 domain highlighting the epitopes (orange) of mAbs H7.HK1 and H7.HK2, with three mutations in red that appeared in the 2016-2017 viral isolates of H7N9. The sticks are interacting CDRs of mAb H7.HK1 heavy and light chains.

Supplementary Table 1 Cryo-EM data collection, refinement, and validation statistics for H7 SH13 DS2 6R HA in complex with H7.HK1 and H7.HK2 Fabs.

	H7 SH13 DS2 6R H7.HK1 (EMD-41422) (PDB: 8TNL)	H7 SH13 DS2 6R H7.HK2 (EMD-41441) (PDB: 8TOA)
**Data collection and processing**		
Magnification	105000	105000
Voltage (kV)	300	300
e–/Å^2	58	58
Defocus range (μm)	0.8-2	0.8-2
Pixel size (Å)	0.83	0.83
Symmetry imposed	C3	C3
Initial particle images (no.)	5713957	2339643
Final particle images (no.)	178347	191469
Map resolution (Å)	3.62	3.69
FSC threshold	0.143	0.143
**Refinement**		
Initial model used (PDB code)	6IDD	8TNL
Model resolution (Å)	3.62	3.69
FSC threshold	0.143	0.143
Model composition		
Non-hydrogen atoms	16487	15570
Protein residues	2112	2109
Ligands	7	11
B factors (Å^2)		
Protein	39	58
Glycans	58	48
R.m.s. deviations		
Bond lengths (Å)	0.005	0.007
Bond angles (°)	1.121	1.231
Validation		
MolProbity score	1.65	2.23
Clashscore	5.45	12.08
Poor rotamers (%)	0.06	1.62
Ramachandran plot		
Favored (%)	94.86	92.30
Allowed (%)	5.14	7.41
Disallowed (%)	0.0	0.29
