# Supplementary material for: Human neutralizing antibodies target a conserved lateral patch on H7N9 hemagglutinin head
Source: Nat Commun. 2024 May 27;15:4505. doi: 10.1038/s41467-024-48758-4 (PMC11130183; doi:10.1038/s41467-024-48758-4)
Supplement: Supplementary file 3 — Reporting Summary [file 41467_2024_48758_MOESM3_ESM.pdf]

nature portfolio

Corresponding author(s): Xueling Wu and Kelvin To

Last updated by author(s): May 6, 2024

Reporting Summary

Nature Portfolio wishes to improve the reproducibility of the work that we publish. This form provides structure for consistency and transparency in reporting. For further information on Nature Portfolio policies, see our Editorial Policies and the Editorial Policy Checklist.

Statistics

For all statistical analyses, confirm that the following items are present in the figure legend, table legend, main text, or Methods section.

n/a	Confirmed	
☐	☒	The exact sample size (*n*) for each experimental group/condition, given as a discrete number and unit of measurement
☐	☒	A statement on whether measurements were taken from distinct samples or whether the same sample was measured repeatedly
☐	☒	The statistical test(s) used AND whether they are one- or two-sided<br>*Only common tests should be described solely by name; describe more complex techniques in the Methods section.*
☒	☐	A description of all covariates tested
☒	☐	A description of any assumptions or corrections, such as tests of normality and adjustment for multiple comparisons
☐	☒	A full description of the statistical parameters including central tendency (e.g. means) or other basic estimates (e.g. regression coefficient) AND variation (e.g. standard deviation) or associated estimates of uncertainty (e.g. confidence intervals)
☐	☒	For null hypothesis testing, the test statistic (e.g. *F*, *t*, *r*) with confidence intervals, effect sizes, degrees of freedom and *P* value noted<br>*Give P values as exact values whenever suitable.*
☒	☐	For Bayesian analysis, information on the choice of priors and Markov chain Monte Carlo settings
☒	☐	For hierarchical and complex designs, identification of the appropriate level for tests and full reporting of outcomes
☒	☐	Estimates of effect sizes (e.g. Cohen's *d*, Pearson's *r*), indicating how they were calculated

Our web collection on statistics for biologists contains articles on many of the points above.

Software and code

Policy information about availability of computer code

Data collection	SoftMax Pro 7.0.2 by Molecular Devices, MoFlo Software by Beckman Coulter
Data analysis	GraphPad Prism 9.5.1; Gene Codes Sequencher 5.4.6; TreeStar FlowJo 10.0; Microsoft 365 Excel version 2404

For manuscripts utilizing custom algorithms or software that are central to the research but not yet described in published literature, software must be made available to editors and reviewers. We strongly encourage code deposition in a community repository (e.g. GitHub). See the Nature Portfolio guidelines for submitting code & software for further information.

Data

Policy information about availability of data

All manuscripts must include a data availability statement. This statement should provide the following information, where applicable:

- Accession codes, unique identifiers, or web links for publicly available datasets
- A description of any restrictions on data availability
- For clinical datasets or third party data, please ensure that the statement adheres to our policy

Sequences of the heavy and light chain variable regions of four H7N9 human mAbs are available in GenBank under accession # OR901962 – OR901969 [https://www.ncbi.nlm.nih.gov/nuccore/OR901962,OR901963,OR901964,OR901965,OR901966,OR901967,OR901968,OR901969]. The Cryo-EM reconstruction of H7.HK1 and H7.HK2 Fabs in complex with H7 SH13 DS2 6R HA has been deposited in the Electron Microscopy Data Bank as EMD-41422 [https://www.ebi.ac.uk/emdb/EMD-41422] and EMD-41441 [https://www.ebi.ac.uk/emdb/EMD-41441] and the Protein Data Bank as PDB: 8TNL [https://www.rcsb.org/structure/8TNL] and 8TOA

[https://www.rcsb.org/structure/8TOA]. Materials will be made available to researchers with appropriate materials transfer agreements (MTAs). All inquiries should be sent to the corresponding authors.

Research involving human participants, their data, or biological material

Policy information about studies with human participants or human data. See also policy information about sex, gender (identity/presentation), and sexual orientation and race, ethnicity and racism.

Reporting on sex and gender	The study subject was a 36-year-old female, which was described in previous publications.
Reporting on race, ethnicity, or other socially relevant groupings	The study subject was an Asian.
Population characteristics	The study subject visited a live poultry market in Shenzhen China in 2013 prior to H7N9 infection.
Recruitment	The study subject was hospitalized for a severe H7N9 2013 infection.
Ethics oversight	Written informed consent was obtained from the study subject. The study was approved by the Institutional Review Board (IRB) of the University of Hong Kong and the Hospital Authority (Reference number: UW-13-265).

Note that full information on the approval of the study protocol must also be provided in the manuscript.

Field-specific reporting

Please select the one below that is the best fit for your research. If you are not sure, read the appropriate sections before making your selection.

☒ Life sciences ☐ Behavioural & social sciences ☐ Ecological, evolutionary & environmental sciences

For a reference copy of the document with all sections, see nature.com/documents/nr-reporting-summary-flat.pdf

Life sciences study design

All studies must disclose on these points even when the disclosure is negative.

Sample size	For animal experiments, 5-15 mice in each group were included for calculating the survival rates and statistical significance. The mouse viral challenge model has been well established and described in previous publications, from which the sample size was known.
Data exclusions	No data was excluded from the analyses.
Replication	Virus neutralization assays were performed in triplicate wells for luciferase readout and in 5 replicate wells for presence of cytopathic effect. Similar results have been independently reproduced at least once. The viral lethal challenge model in mice was replicated successfully for more than 3 times through out the study.
Randomization	Mice of 6-8 weeks of age were randomized into experimental groups.
Blinding	The investigators were blinded to animal group allocation during data collection.

Reporting for specific materials, systems and methods

We require information from authors about some types of materials, experimental systems and methods used in many studies. Here, indicate whether each material, system or method listed is relevant to your study. If you are not sure if a list item applies to your research, read the appropriate section before selecting a response.

Materials & experimental systems

n/a	Involved in the study	
☐	☒	Antibodies
☐	☒	Eukaryotic cell lines
☒	☐	Palaeontology and archaeology
☐	☒	Animals and other organisms
☒	☐	Clinical data
☒	☐	Dual use research of concern
☒	☐	Plants

Methods

n/a	Involved in the study	
☒	☐	ChIP-seq
☐	☒	Flow cytometry
☒	☐	MRI-based neuroimaging

Antibodies

Antibodies used	CD3-PE-CF594 (BD Biosciences, Cat. No. 562406, Clone SP34-2), CD19-PE-Cy7 (BioLegend, Cat. No. 302216, Clone HIB19), CD20-APC-Cy7 (BioLegend, Cat. No. 302314, Clone 2H7), IgG-FITC (BD Biosciences, Cat. No. 555786, Clone G18-145), IgM-V450 (BD Biosciences, Cat. No. 561286, Clone G20-127), Horseradish peroxidase (HRP)-conjugated goat anti-human IgG Fc (Jackson ImmunoResearch, Cat. No. 109-035-098).
Validation	BD Biosciences, BioLegend, and Jackson ImmunoResearch provided Quality Certificates for their antibody products. All staining antibodies were further validated using healthy human blood donor PBMCs purchased from the New York Blood Center.

Eukaryotic cell lines

Policy information about cell lines and Sex and Gender in Research

Cell line source(s)	Human embryonic kidney 293 cell line, of which the sex is female, is the parental cell for 293T and Expi293F. 293T was obtained from ATCC (Cat. No. CRL-11268, Clone 17). Expi293F was obtained from ThermoFisher Scientific (Cat. No. A14527). Madin-Darby Canine Kidney (MDCK) cell line, of which the sex is female, was obtained from ATCC (Cat. No. CCL-34).
Authentication	The vendors provided Certificate of analysis for their cell lines. No further authentication was performed on cell lines used in this study.
Mycoplasma contamination	The cell lines were not tested for mycoplasma contamination, which was not evident during the study.
Commonly misidentified lines (See ICLAC register)	None.

Animals and other research organisms

Policy information about studies involving animals; ARRIVE guidelines recommended for reporting animal research, and Sex and Gender in Research

Laboratory animals	BALB/C female mice (6-8 weeks old) were used.
Wild animals	The study did not involve wild animals.
Reporting on sex	Female mice were used because the viral challenge model was previously established with only female mice.
Field-collected samples	The study did not involve samples collected from the field.
Ethics oversight	The mouse studies were approved by the Committee on the Use of Live Animals in Teaching and Research (CULATR) of the University of Hong Kong (Reference number: 4011-16).

Note that full information on the approval of the study protocol must also be provided in the manuscript.

Plants

Seed stocks	*Report on the source of all seed stocks or other plant material used. If applicable, state the seed stock centre and catalogue number. If plant specimens were collected from the field, describe the collection location, date and sampling procedures.*
Novel plant genotypes	*Describe the methods by which all novel plant genotypes were produced. This includes those generated by transgenic approaches, gene editing, chemical/radiation-based mutagenesis and hybridization. For transgenic lines, describe the transformation method, the number of independent lines analyzed and the generation upon which experiments were performed. For gene-edited lines, describe the editor used, the endogenous sequence targeted for editing, the targeting guide RNA sequence (if applicable) and how the editor was applied.*
Authentication	*Describe any authentication procedures for each seed stock used or novel genotype generated. Describe any experiments used to assess the effect of a mutation and, where applicable, how potential secondary effects (e.g. second site T-DNA insertions, mosiacism, off-target gene editing) were examined.*

Flow Cytometry

Plots

Confirm that:

☒ The axis labels state the marker and fluorochrome used (e.g. CD4-FITC).

☒ The axis scales are clearly visible. Include numbers along axes only for bottom left plot of group (a 'group' is an analysis of identical markers).

☒ All plots are contour plots with outliers or pseudocolor plots.

☒ A numerical value for number of cells or percentage (with statistics) is provided.

Methodology

Sample preparation	Human PBMCs were stained with an antibody cocktail to cell markers. Live/dead yellow stain (Invitrogen, Cat. No. L34968) was used to exclude dead cells.
Instrument	MoFlo sorter by Beckman Coulter
Software	MoFlo software by Beckman Coulter for data collection, TreeStar FlowJo 10.0 for data analysis
Cell population abundance	Sorted single cell was not feasible to check purity by flow, but the target genes were amplified post-sort by single cell RT-PCR.
Gating strategy	FSC-A 75K~170K and SSC-A 25K~75K to gate lymphocytes, followed by live/dead yellow negative, CD3-PE-CF594 negative, CD19-PE-Cy7 positive, CD20-APC-Cy7 positive and negative, IgG-FITC positive, IgM-V450 positive and negative, H7-PE positive.

☐ Tick this box to confirm that a figure exemplifying the gating strategy is provided in the Supplementary Information.
